# Lacosamide and Levetiracetam Have No Effect on Sharp-Wave Ripple Rate

**DOI:** 10.3389/fneur.2017.00687

**Published:** 2017-12-21

**Authors:** Jan Kudlacek, Jan Chvojka, Antonin Posusta, Lubica Kovacova, Seung Bong Hong, Shennan Weiss, Kamila Volna, Petr Marusic, Jakub Otahal, Premysl Jiruska

**Affiliations:** ^1^Department of Developmental Epileptology, Institute of Physiology, The Czech Academy of Sciences, Prague, Czechia; ^2^Department of Circuit Theory, Faculty of Electrical Engineering, Czech Technical University in Prague, Prague, Czechia; ^3^Department of Neurology, Samsung Medical Center, Samsung Advanced Institute for Health Sciences & Technology (SAIHST), Sungkyunkwan University School of Medicine, Seoul, South Korea; ^4^Samsung Biomedical Research Institute, Seoul, South Korea; ^5^Department of Neurology, Thomas Jefferson University, Philadelphia, PA, United States; ^6^Department of Neurology, 2nd Faculty of Medicine, Charles University and Motol University Hospital, Prague, Czechia

**Keywords:** high-frequency oscillations, sharp-wave ripples, levetiracetam, lacosamide, antiepileptic drugs, hippocampus, ripples, *in vivo*

## Abstract

Pathological high-frequency oscillations are a novel marker used to improve the delineation of epileptogenic tissue and, hence, the outcome of epilepsy surgery. Their practical clinical utilization is curtailed by the inability to discriminate them from physiological oscillations due to frequency overlap. Although it is well documented that pathological HFOs are suppressed by antiepileptic drugs (AEDs), the effect of AEDs on normal HFOs is not well known. In this experimental study, we have explored whether physiological HFOs (sharp-wave ripples) of hippocampal origin respond to AED treatment. The results show that application of a single dose of levetiracetam or lacosamide does not reduce the rate of sharp-wave ripples. In addition, it seems that these new generation drugs do not negatively affect the cellular and network mechanisms involved in sharp-wave ripple generation, which may provide a plausible explanation for the absence of significant negative effects on cognitive functions of these drugs, particularly on memory.

## Highlights

- Pathological high-frequency oscillations (pHFOs) represent electrographic biomarker of epileptogenic tissue.
- Current approaches are not able to distinguish pathological HFOs from physiological ones in intracranial recordings.
- Antiepileptic drugs decrease the rate of pHFOs.
- Levetiracetam or lacosamide do not decrease the rate of sharp-wave ripples—a representative of physiological HFOs.
- Pharmacological testing could be used to discriminate pathological and physiological HFOs.

## Introduction

Pathological high-frequency oscillations (pHFOs) represent a new electrographic marker of epileptogenic tissue. Early after their discovery, pHFO analysis was introduced into the presurgical evaluation to better delineate the resection margin and to improve the outcome of surgery. pHFOs are classified according to their frequency into two main groups—ripples (80–250 Hz) and fast ripples (250–600 Hz) (1, 2). Although fast ripples are considered more specific for epileptogenic tissue than ripples, both types of pHFO can localize the epileptogenic zone or seizure onset areas in humans who undergo exploration with invasive electrodes (1, 2). Several studies have demonstrated the beneficial effect of complete resection of the pHFO generating areas on surgical outcome (3–5). The practical utilization of pHFOs in presurgical evaluation is substantially hindered by the inability to differentiate them from physiological high-frequency oscillations, such as hippocampal sharp-wave ripples (SWRs) as they display substantial frequency overlap (2, 6, 7). Currently, we do not have any effective tools, which can reliably discriminate between them. Identification of an approach to reliably discriminate between normal and pathological HFOs is a complex, but essential, issue to address if the properties of pHFOs are to be fully utilized in clinical practice.

One of the features of pHFOs is their responsiveness to antiepileptic drugs (AEDs). In the chronic pilocarpine model of temporal lobe epilepsy, pHFOs and seizure rate decrease after treatment with levetiracetam (8) or lacosamide (9). In humans, withdrawal of AEDs is associated with increased rate of pHFOs (10). A pharmacological test using AEDs could be a plausible strategy to discriminate physiological oscillations from pathological ones, providing rate of physiological HFOs is not decreased by AEDs.

In this proof-of-principle study, we explored the impact of a single dose of lacosamide or levetiracetam on the rate of SWRs—a hippocampal representative of physiological HFOs (11). SWRs play a crucial role in the process of coordinated memory reactivation and formation of long-term memory (12). We have tested the hypothesis that the application of a single therapeutic dose of levetiracetam or lacosamide does not affect SWR rate.

## Materials and Methods

### Electrode Implantation and EEG Recording

**Figure 1:**
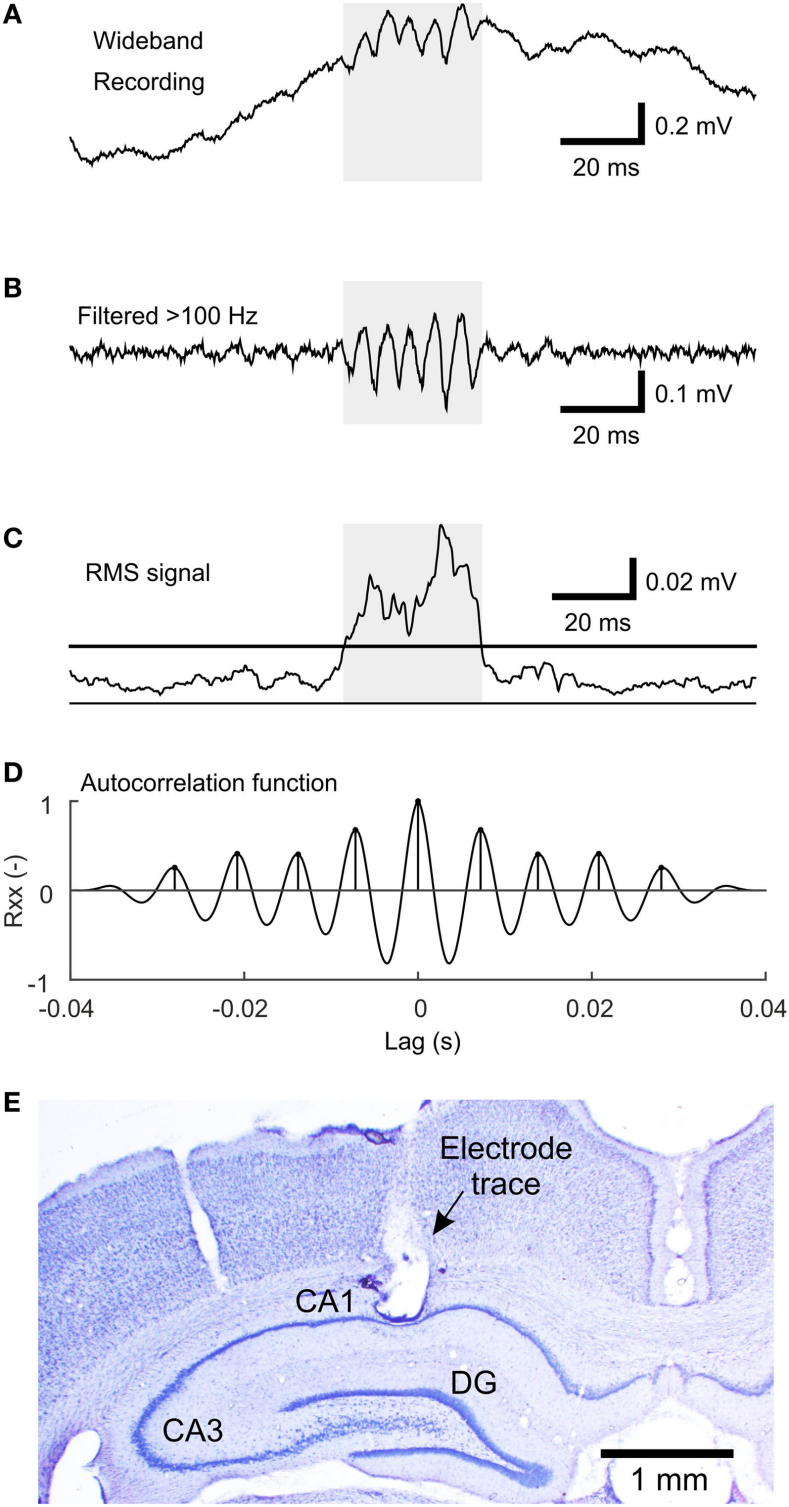
The algorithm of sharp-wave ripple (SWR) detection. **(A)** An example of the wideband signal containing an SWR event. **(B)** High-pass filtered (>100 Hz) signal reveals the ripple oscillation. **(C)** Root mean square (RMS) signal computed from the high-pass filtered signal. The line indicates the detection threshold. The shaded area marks the putative detection. **(D)** The autocorrelation function of the shaded segment of the high-pass filtered signal with detected peaks, which fulfills the criteria for SWR detection. **(E)** An example of a Nissl-stained brain slice with electrode tip located in stratum pyramidale of the CA1 hippocampal subregion.

All experiments were performed under the Animal Care and Animal Protection Law of the Czech Republic fully compatible with the guidelines of the European Union directive 2010/63/EU. The protocol was approved by the Ethics Committee of The Czech Academy of Sciences (Project License No. 71/2016). Animals were housed in groups under standard conditions in a room with controlled temperature (22 ± 1°C) and 12/12 h light/dark cycle. Eleven adult male Wistar rats weighing between 350 and 430 g were used in this study. The surgical preparation was performed under isoflurane anesthesia. The animals were implanted with bipolar twisted silver electrodes (120 µm in diameter, AM Systems, Inc., USA) bilaterally in the stratum pyramidale (AP: −4.1, L: 2.2, D: 2.5) and stratum radiatum (AP: −4.6, L: 2.6, D: 2.6) of the dorsal CA1 according to the stereotaxic atlas (13). The two contacts of each electrode were 0.5 mm apart. Two ground/reference jeweler’s screws were placed over the cerebellum. Following a 5-day recovery period, animals were individually video-EEG monitored for 4 weeks continuously. Spontaneous electrographic activity was amplified, band-pass filtered (0.1 Hz–1.6 kHz), and digitized at 5 kHz using a RHD2132 32-channel amplifier chip (Intan Technologies, USA). After the end of the experiment, animals were humanely euthanized by an overdose of urethane, brains extracted, and processed to verify the positions of electrodes (Figure 1E).

### AED Treatment

Each animal received sequential intraperitoneal injections of levetiracetam (Keppra^®^, UCB, S.A., Brussels, Belgium), saline (control solution) of equal volume, lacosamide (Vimpat^®^, UCB, S.A., Brussels, Belgium), and saline of equal volume. Additionally, three of the animals received diazepam (Apaurin^®^, Krka, d. d., Novo Mesto, Slovenia) and saline of equal volume. Diazepam acted as a positive control since it was shown to decrease the SWR rate *in vivo* (14). The interval after each injection, whether it was an AED or saline, was 3 days to allow maximal elimination of the drug from the body based on the known pharmacokinetics (14–16). The doses for levetiracetam and lacosamide were 80 and 30 mg/kg, respectively. At comparable doses, these drugs were shown to effectively suppress pHFOs (8, 9). The dose of diazepam was 1 mg/kg which was shown to reduce or even suppress the SWR rate (14, 17). The sequence of injections was randomized between animals. In a given rat, every injection was administered at the same time of the day (10:30 a.m. or 2:00 p.m.).

### EEG Analysis

EEGs were analyzed using custom made scripts in Matlab 2015a computing environment (Mathworks Inc., Natick, MA, USA). Two animals were removed from the analysis due to extremely noisy EEG signals. In one animal, the experiment was terminated prematurely due to the loss of the head cap prior to lacosamide injection. Therefore, the total number of animals used in the evaluation of the three drugs was nine for levetiracetam, eight for lacosamide, and three for diazepam. In each animal, SWRs were analyzed only in the channel in which SWRs displayed the highest amplitude. Correct positions of these electrodes in the hippocampal CA1 were verified histologically. We analyzed two epochs each lasting 2 h. The first epoch was from 0.5 to 2.5 h after the injection and the second one was from 4.5 to 6.5 h after the injection. To determine the SWR rate during the same brain state, we extracted episodes of slow-wave sleep only, from each epoch, based on the presence of slow waves in the EEG and verified behaviorally in video recordings.

### SWR Detection

Sharp-wave ripples were detected using the modified root mean square (RMS)-based approach (18). The signal was band-pass filtered (passband 100–200 Hz) using a FIR filter with a 40-Hz wide transition band and stopband attenuation at 80 dB. RMS value was calculated in 4 ms sliding windows. Only segments of at least 18 ms in duration with an RMS value >1.5 SD above the RMS mean were selected as putative SWRs. Events closer than 10 ms were treated as a single event. The next step of the detection procedure included estimation of the autocorrelation function in high-pass filtered segments (>100 Hz, otherwise the same parameters). Detections with at least seven peaks in the autocorrelation function with a lag corresponding to the SWR frequency band (100–200 Hz) and with the second peak of at least 25% of magnitude were selected. The main steps of the detection procedure are visualized in Figures 1A–D.

To evaluate the detector’s performance, 24 randomly selected epochs of slow-wave sleep were labeled by an expert. Automatic detections were compared to the expert’s labels. A true positive (TP) detection was defined as the one overlapping with an expert’s label by at least 50% of the detection’s duration. A false positive (FP) detection was a detection not fulfilling the criteria for TP detection. The number of false negatives (FN) was defined as the number of expert’s labels, which had no overlap with any of the TP detections. Finally, sensitivity and positive predictive value (PPV) of the detector was calculated using the following equations:

$$\text{sensitivity}=\frac{\text{TP}}{\text{TP}+\text{FN}}$$

$$\text{PPV}=\frac{\text{TP}}{\text{TP}+\text{FP}}.$$

Sensitivity and PPV of the detector was 57 and 80%, respectively. Detector’s settings were optimized to achieve mainly high PPV to detect only true SWRs, omit ambiguous SWRs, and to minimize the risk of FP detections.

### Statistical Evaluation

In this study, the primary hypothesis tested was that injections of selected AEDs do not affect SWR rates. This is in contrast to the vast majority of drug studies, which examine and test for the presence of an effect of the drug on specific phenomena, including HFOs. Thus, we had to implement an appropriate statistical method that tests equality and not an effect. In this study, we adopted the method recommended by Piaggio et al. (19). For each rat, each injection and each time after the injection we calculated average SWR rate during the slow-wave sleep epochs. Then, for each rat, each drug and each time after the injection, we calculated the ratio of SWR rate after AED injection to SWR rate after the corresponding saline injection. Since the data did not display normal distribution, we used a non-parametric approach. For each drug and each time after the injection, median of the ratios and its non-parametric confidence interval was calculated. The non-parametric confidence interval for the median is obtained as the *k*-th lowest and the *k*-th highest value from the sample. *k* is determined so that the true population median lies within that interval with confidence equal or higher than required. In our study, we required at least 90% confidence. For sample sizes nine and eight we took *k* = 2 which gives confidences of 96 and 93%, respectively. For the three diazepam animals, we took the first and the last value as the confidence interval (widest possible), which gives up to 75% confidence (20). We obtained confidence intervals for all three drugs and two time windows after the injections. These six confidence intervals were compared to equality margins, which were set to 0.75 and 1.25. If the confidence interval did not cross the equality margins, the AED was considered to have no effect on SWR rate.

Following equality testing, we performed statistical analysis to evaluate the possible presence of a statistically significant effect of AEDs on the SWR rate. In this step, we used the Wilcoxon signed-rank test on each set of the ratios. Before applying the test, the ratios were transformed by subtracting 1 so that a decrease in the SWR rate resulted in a negative number and *vice versa*. In the case of a non-significant result, *post hoc* power of the Wilcoxon signed-rank test was determined using SD of the data and location shift equal to the equality margin, i.e., 0.25 (21, 22).

## Results

**Figure 2:**
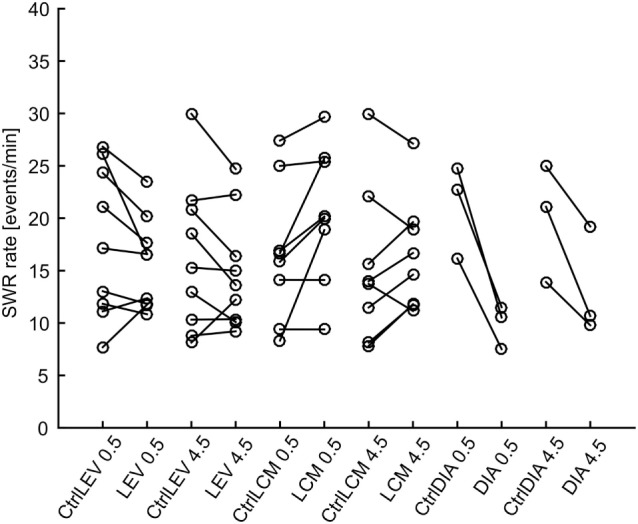
Sharp-wave ripple (SWR) rates after various treatments. Lines connect data points from individual animals. LEV, levetiracetam; LCM, lacosamide; DIA, diazepam; CtrlXXX, injection of equivalent volume of saline; 0.5, half an hour after injection, 4.5, four and half hours after injection.

**Figure 3:**
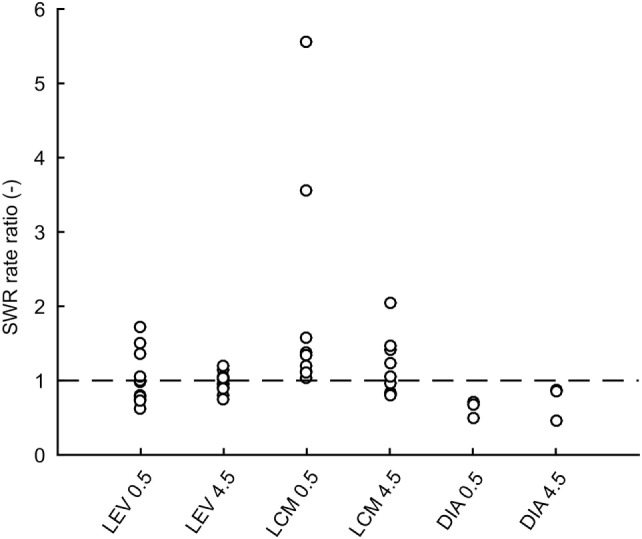
Ratios of sharp-wave ripple (SWR) rates after drug treatment to rates after saline treatment for individual rats. Dashed line is at value 1 which constitutes no effect. LEV, levetiracetam; LCM, lacosamide; DIA, diazepam; CtrlXXX, injection of an equivalent volume of saline; 0.5, half an hour after injection; 4.5, four and half hours after injection.

**Figure 4:**
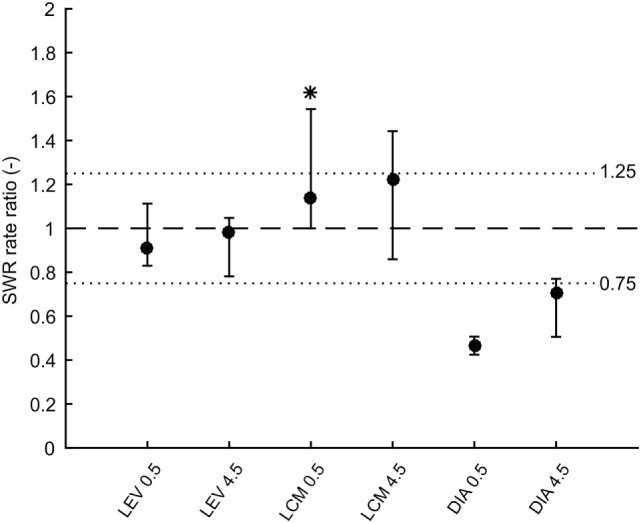
Medians of ratios of sharp-wave ripple (SWR) rates after drug injection to rates after corresponding saline injection. Error bars represent non-parametric confidence intervals of the medians. Dashed line is at value 1 which constitutes no effect. Dotted lines at values 0.75 and 1.25 represent equality margins. LEV confidence intervals are at 96% confidence and do not cross the equality margins. LCM significantly increases SWR rate 0.5 h after administration, but 4.5 h after administration the effect dissipates. Confidence intervals are 93%. DIA markedly reduces SWR rate 0.5 h after administration and 4.5 h after administration the effect slowly dissipates. Confidence intervals are 75%. LEV, levetiracetam; LCM, lacosamide; DIA, diazepam.

In total 43,011 SWRs were detected with 4,779 ± 3,125 events per animal. The average SWR rate was 16.0 events/min, which is congruent with studies focused on SWR *in vivo* (11, 23–25). SWR rates of all animals, after each injection, in both analyzed time windows, are shown in Figure 2. The data revealed the presence of individual variability in the SWR rate and response to the tested drugs. The crucial parameter for examination of the effect of an AED is the ratio between the SWR rate after AED injection and the SWR rate after control saline injection (Figure 3). The median ratio of the SWR rate between levetiracetam and the control was 0.91 and 0.98 for 30 min and 4.5 h after the injection, respectively (*n* = 9 animals). For levetiracetam, the ratios’ confidence intervals did not cross the equality margins 30 min and 4.5 h after injection (Figure 4). Therefore, SWR rates after levetiracetam and saline treatment can be considered equal within the equality margins. The Wilcoxon signed-rank test for ratios was non-significant for 30 min (*p* = 0.30) and 4.5 h after the injection (*p* = 0.57), with a *post hoc* power of 88%. For lacosamide, the median ratio of the SWR rate was 1.14 and 1.22 for each time epoch (*n* = 8 animals). The confidence intervals of the SWR rate ratios crossed the equality margins in both time windows. The Wilcoxon signed-rank test demonstrated a significant increase in SWR rate ratio 30 min after injection of lacosamide (*p* = 0.039). After 4.5 h the effect of lacosamide was non-significant (Wilcoxon signed-rank test; *p* = 0.11; power = 85%). Diazepam, which was used as a positive control, reduced the SWR rate compared to the equivalent volume of saline in all rats by >50% with a median ratio of 0.46 and 0.70 for each time window (Figure 4). However, statistical significance could not be reached due to the small number of rats (*n* = 3 animals; Wilcoxon signed-rank test; *p* = 0.25; power = 46%).

## Discussion

We have shown that a single dose of levetiracetam and lacosamide does not reduce the rate of SWRs—a representative of physiological HFOs. The ability to reliably differentiate between pathological and physiological HFOs represents a crucial step toward the clinical utilization of pHFOs as biomarkers of the epileptogenic zone (2, 7). Epileptic pHFOs display a spectral overlap with physiological HFOs such as SWRs or fast gamma activity. Fast ripples are considered to be exclusively of epileptic origin, but physiological activity with a frequency around ~600 Hz has been described in the neocortex. Matsumoto and colleagues described features, which discriminated task-related physiological HFOs from pathological ones (26). Pathological HFOs were characterized by a higher spectral mean, longer duration, and lower mean frequency. Other studies tried to discriminate HFOs according to their spatial distribution, relationship with sleep phases (27), background activity (28), phase relationship with slow waves (29), or using cognitive tasks (30). However, the practical implementation of these criteria is challenging. Experimental treatment with levetiracetam or lacosamide has been shown to reduce the both ripples and fast ripples in the pilocarpine model of temporal lobe epilepsy, even though the effects were region specific (8, 9). In this *in vivo* study, we demonstrate that levetiracetam does not decrease the rate of hippocampal SWRs. The confidence intervals were set to tolerate a 25% change in SWR rate after the treatment. Studies which explored the effect of levetiracetam and lacosamide on pHFOs showed that these drugs reduced the pathological ripple rate in hippocampal structures by an average of 57% for levetiracetam and by 43% for lacosamide (8, 9). Although we studied the effect of these AEDs only on specific subtype of physiological HFOs, these experimental results suggest that the procedure of pharmacological testing can be a plausible approach to facilitate discrimination between physiological and pathological HFOs. Both drugs are used very frequently and their withdrawal and subsequent introduction is a very common procedure in patients undergoing invasive explorations. As an alternative, the introduction of AEDs could be replaced by an intravenous application of levetiracetam at the end of monitoring. HFOs that are not altered by AEDs can be considered to be physiological HFOs.

The absence of a major effect of the AEDs on the rate of SWRs and suppression of pHFOs can be explained by the currently known mechanisms of action of the drugs and cellular mechanisms of HFOs (31, 32). SWRs are oscillations that reflect the activation of large neuronal ensembles in the hippocampal CA1 region, particularly during sleep. On the cellular level, the oscillation depends on the precise interaction between pyramidal cells and inhibitory interneurons. The fast inhibitory postsynaptic potentials on the membrane of principal neurons play a key role in the coordination of principal cell firing (11). Pyramidal cells fire heterogeneously during the SWR; some cells can fire during every successive event, while other cells can fire only occasionally (17, 33). The sequence of neuronal activity during SWRs replays the sequence of neuronal activation during behavioral tasks, and it is considered to represent a mechanism of memory reactivation and long-term memory formation (12, 34). The cellular dynamics of pHFOs in both ripple and fast ripple bands differs and is more uniform (31, 32). During each pHFO, a large population of cells generates a burst of high-frequency action potentials superimposed on a large depolarizing envelope (35–37). While high-frequency spiking depends mainly on fast sodium channel kinetics, the depolarizing envelope is associated with an increase in intracellular calcium *via* the opening of voltage-gated calcium channels or activation of non-NMDA and NMDA receptors. Lacosamide modifies voltage-gated sodium channel kinetics required for fast action potential firing, and it affects only neurons which are active or depolarized for prolonged periods of time and thus spares physiological functions (38). Therefore, pathological pHFOs are more susceptible to its effects than SWRs. The levetiracetam binds to SV2A, which is involved in trafficking and fusion of synaptic vesicles (39). It seems that levetiracetam reduces the vesicle release that is important for synaptic neurotransmission (40). It also partially blocks N-type calcium channels (41). However, the exact mechanisms of its actions are not well known, but we can assume that levetiracetam is capable of interfering with cellular processes involved in intense neuronal firing and pHFOs.

The absence of the effect of levetiracetam and lacosamide on the mechanisms of SWRs, in general, can also be deduced indirectly from the lack of their negative effect on cognitive functions including memory. It is well established that any drug or procedure, which has the capacity to interfere with the cellular mechanisms underlying the genesis of SWRs, also has the capacity to induce a memory deficit (42, 43). Levetiracetam is currently the drug of first choice, and lacosamide has also shown its therapeutic benefits as add-on therapy early after its introduction into epilepsy therapy. Both drugs are well tolerated by patients and demonstrate low adverse effects and an absence of a significant impact on cognition and memory (44–47). From electrophysiological perspective, these observations are supported by our experimental study, which demonstrates the absence of any suppressive effect of these drugs on the SWR generation. Moreover, lacosamide was shown to increase the speed of complex visual information processing (48), which may be in agreement with the transiently increased SWR rate observed in this study.

A possible limitation of this study is the single-dose scheme of drug administration. We might have missed the time period of SWR suppression by the drug. However, based on the known pharmacokinetics and mechanism of action of these drugs this seems unlikely (15, 16, 38, 49). Another possibility is that the drug was cleared from the body before its concentration in the brain could have reached sufficient intrathecal levels to influence the SWR rate. However, we used the same dose that was administered daily in studies exploring the effect of lacosamide and levetiracetam on pHFOs (8, 9) and pharmacokinetic studies showed that these drugs reach maximal brain concentration within 2 h after application (15, 16). Another weakness of the study is the single-dose application. We cannot exclude that prolonged or chronic application of these drugs may induce long-term changes, which would affect the properties of SWRs.

In conclusion, we have shown that levetiracetam does not change the rate of SWRs and lacosamide transiently increases it. Hence, these drugs can be considered for pharmacological testing to distinguish physiological versus pHFOs. Levetiracetam or lacosamide could be introduced toward the end of invasive exploration, and the response of HFOs to the drug introduction could be used to determine whether the HFOs are pathological or physiological. Moreover, our results are congruent with studies showing no negative effect of these drugs on cognition.

## Ethics Statement

This study was carried out in accordance with the recommendations of the Animal Care and Animal Protection Law of the Czech Republic fully compatible with the guidelines of the European Union directive 2010/63/EU. The protocol was approved by the Ethics Committee of The Czech Academy of Sciences (Project License No. 71/2016).

## Author Contributions

Conceived and designed the experiments: PJ, JO, PM, and SH. Performed the experiments: JC, JK, and LK. Analyzed the data: JC, JK, LK, AP, PJ, and JO. Wrote the paper: JC, JK, PJ, KV, SW, PM, JO, and SH.

## Conflict of Interest Statement

PM has received honoraria from UCB as a speaker and advisory board member. The remaining authors have no conflicts of interest. We confirm that we have read the Journal’s position on issues involved in ethical publication and affirm that this report is consistent with those guidelines.

## References

[1] Zijlmans M Jiruska P Zelmann R Leijten FS Jefferys JG Gotman J High-frequency oscillations as a new biomarker in epilepsy. Ann Neurol (2012) 71:169–78. 10.1002/ana.22548 22367988 PMC3754947

[2] Frauscher B Bartolomei F Kobayashi K Cimbalnik J van ’t Klooster MA Rampp S High-frequency oscillations: the state of clinical research. Epilepsia (2017) 58:1316–29. 10.1111/epi.13829. 28666056 PMC5806699

[3] Jacobs J Zijlmans M Zelmann R Chatillon CE Hall J Olivier A High-frequency electroencephalographic oscillations correlate with outcome of epilepsy surgery. Ann Neurol (2010) 67:209–20. 10.1002/ana.21847 20225281 PMC3769290

[4] Cho JR Koo DL Joo EY Seo DW Hong SC Jiruska P Resection of individually identified high-rate high-frequency oscillations region is associated with favorable outcome in neocortical epilepsy. Epilepsia (2014) 55:1872–83. 10.1111/epi.12808 25266626

[5] Holler Y Kutil R Klaffenbock L Thomschewski A Holler PM Bathke AC High-frequency oscillations in epilepsy and surgical outcome. A meta-analysis. Front Hum Neurosci (2015) 9:574. 10.3389/fnhum.2015.00574 26539097 PMC4611152

[6] Engel J Jr Bragin A Staba R Mody I. High-frequency oscillations: what is normal and what is not? Epilepsia (2009) 50:598–604. 10.1111/j.1528-1167.2008.01917.x 19055491

[7] Cimbalnik J Kucewicz MT Worrell G. Interictal high-frequency oscillations in focal human epilepsy. Curr Opin Neurol (2016) 29:175–81. 10.1097/WCO.0000000000000302 26953850 PMC4941960

[8] Levesque M Behr C Avoli M. The anti-ictogenic effects of levetiracetam are mirrored by interictal spiking and high-frequency oscillation changes in a model of temporal lobe epilepsy. Seizure (2015) 25:18–25. 10.1016/j.seizure.2014.11.008 25645630 PMC4880465

[9] Behr C Levesque M Ragsdale D Avoli M Lacosamide modulates interictal spiking and high-frequency oscillations in a model of mesial temporal lobe epilepsy. Epilepsy Res (2015) 115:8–16. 10.1016/j.eplepsyres.2015.05.006 26220372 PMC4878889

[10] Zijlmans M Jacobs J Zelmann R Dubeau F Gotman J. High-frequency oscillations mirror disease activity in patients with epilepsy. Neurology (2009) 72:979–86. 10.1212/01.wnl.0000344402.20334.81 19289737 PMC3797085

[11] Ylinen A Bragin A Nadasdy Z Jando G Szabo I Sik A Sharp wave-associated high-frequency oscillation (200 Hz) in the intact hippocampus: network and intracellular mechanisms. J Neurosci (1995) 15:30–46. 7823136 10.1523/JNEUROSCI.15-01-00030.1995 PMC6578299

[12] Buzsáki G. Hippocampal sharp wave-ripple: a cognitive biomarker for episodic memory and planning. Hippocampus (2015) 25:1073–188. 10.1002/hipo.22488 26135716 PMC4648295

[13] Paxinos G Watson C The rat brain. 4th ed The Rat Brain in Stereotaxic Coordinates. San Diego, CA: Academic Press, Inc (1998). 474 p.

[14] Ponomarenko AA Korotkova TM Sergeeva OA Haas HL. Multiple GABAA receptor subtypes regulate hippocampal ripple oscillations. Eur J Neurosci (2004) 20:2141–8. 10.1111/j.1460-9568.2004.03685.x 15450093

[15] Tong X Patsalos PN. A microdialysis study of the novel antiepileptic drug levetiracetam: extracellular pharmacokinetics and effect on taurine in rat brain. Br J Pharmacol (2001) 133:867–74. 10.1038/sj.bjp.0704141 11454660 PMC1572849

[16] Koo TS Kim SJ Ha DJ Baek M Moon H. Pharmacokinetics, brain distribution, and plasma protein binding of the antiepileptic drug lacosamide in rats. Arch Pharm Res (2011) 34:2059–64. 10.1007/s12272-011-1208-7 22210031

[17] Buzsaki G Horvath Z Urioste R Hetke J Wise K. High-frequency network oscillation in the hippocampus. Science (1992) 256:1025–7. 10.1126/science.1589772 1589772

[18] Staba RJ Wilson CL Bragin A Fried I Engel J Jr. Quantitative analysis of high-frequency oscillations (80–500 Hz) recorded in human epileptic hippocampus and entorhinal cortex. J Neurophysiol (2002) 88:1743–52. 10.1152/jn.00322.2002 12364503

[19] Piaggio G Elbourne DR Pocock SJ Evans SJ Altman DG CONSORT Group. Reporting of noninferiority and equivalence randomized trials: extension of the CONSORT 2010 statement. JAMA (2012) 308:2594–604. 10.1001/jama.2012.87802 23268518

[20] Hogg RV Tanis EA Probability and Statistical Inference. New Jersey: Prentice Hall (2006).

[21] Lehmann EL Nonparametrics: Statistical Methods Based on Ranks. San Francisco: Holden-day, Inc. (1975).

[22] Shieh G Jan S-L Randles RH Power and sample size determinations for the Wilcoxon signed-rank test. J Stat Comput Simul (2007) 77:717–24. 10.1080/10629360600635245

[23] Csicsvari J Hirase H Czurko A Mamiya A Buzsaki G Fast network oscillations in the hippocampal CA1 region of the behaving rat. J Neurosci (1999) 19:Rc20. 10436076 10.1523/JNEUROSCI.19-16-j0001.1999 PMC6782850

[24] Eschenko O Ramadan W Mölle M Born J Sara SJ. Sustained increase in hippocampal sharp-wave ripple activity during slow-wave sleep after learning. Learn Mem (2008) 15:222–8. 10.1101/lm.726008 18385477 PMC2327264

[25] Cheng S Frank LM. New experiences enhance coordinated neural activity in the hippocampus. Neuron (2008) 57:303–13. 10.1016/j.neuron.2007.11.035 18215626 PMC2244590

[26] Matsumoto A Brinkmann BH Matthew Stead S Matsumoto J Kucewicz MT Marsh WR Pathological and physiological high-frequency oscillations in focal human epilepsy. J Neurophysiol (2013) 110:1958–64. 10.1152/jn.00341.2013 23926038 PMC3798937

[27] Frauscher B von Ellenrieder N Dubeau F Gotman J. EEG desynchronization during phasic REM sleep suppresses interictal epileptic activity in humans. Epilepsia (2016) 57:879–88. 10.1111/epi.13389 27112123 PMC4949560

[28] Kerber K Dumpelmann M Schelter B Le Van P Korinthenberg R Schulze-Bonhage A Differentiation of specific ripple patterns helps to identify epileptogenic areas for surgical procedures. Neurophysiol Clin (2014) 125:1339–45. 10.1016/j.clinph.2013.11.030 24368032

[29] von Ellenrieder N Frauscher B Dubeau F Gotman J Interaction with slow waves during sleep improves discrimination of physiologic and pathologic high-frequency oscillations (80–500 Hz). Epilepsia (2016) 57:869–78. 10.1111/epi.13380 27184021

[30] Axmacher N Elger CE Fell J. Ripples in the medial temporal lobe are relevant for human memory consolidation. Brain (2008) 131:1806–17. 10.1093/brain/awn103 18503077

[31] Jefferys JG Menendez de la PL Wendling F Bragin A Avoli M Timofeev I Mechanisms of physiological and epileptic HFO generation. Prog Neurobiol (2012) 98:250–64. 10.1016/j.pneurobio.2012.02.005 22420980 PMC4873284

[32] Jiruska P Alvarado-Rojas C Schevon CA Staba R Stacey W Wendling F Update on the mechanisms and roles of high-frequency oscillations in seizures and epileptic disorders. Epilepsia (2017) 58:1330–9. 10.1111/epi.13830 28681378 PMC5554080

[33] Csicsvari J Hirase H Czurko A Mamiya A Buzsaki G Oscillatory coupling of hippocampal pyramidal cells and interneurons in the behaving rat. J Neurosci (1999) 19:274–87. 9870957 10.1523/JNEUROSCI.19-01-00274.1999 PMC6782375

[34] Csicsvari J O’Neill J Allen K Senior T. Place-selective firing contributes to the reverse-order reactivation of CA1 pyramidal cells during sharp waves in open-field exploration. Eur J Neurosci (2007) 26:704–16. 10.1111/j.1460-9568.2007.05684.x 17651429 PMC2121123

[35] Bragin A Wilson CL Engel J Jr. Chronic epileptogenesis requires development of a network of pathologically interconnected neuron clusters: a hypothesis. Epilepsia (2000) 41(Suppl 6):S144–52. 10.1111/j.1528-1157.2000.tb01573.x 10999536

[36] Foffani G Uzcategui YG Gal B Menendez de la PL. Reduced spike-timing reliability correlates with the emergence of fast ripples in the rat epileptic hippocampus. Neuron (2007) 55:930–41. 10.1016/j.neuron.2007.07.040 17880896

[37] Ibarz JM Foffani G Cid E Inostroza M Menendez de la PL. Emergent dynamics of fast ripples in the epileptic hippocampus. J Neurosci (2010) 30:16249–61. 10.1523/JNEUROSCI.3357-10.2010 21123571 PMC6634823

[38] Rogawski MA Tofighy A White HS Matagne A Wolff C. Current understanding of the mechanism of action of the antiepileptic drug lacosamide. Epilepsy Res (2015) 110:189–205. 10.1016/j.eplepsyres.2014.11.021 25616473 PMC13325623

[39] Lynch BA Lambeng N Nocka K Kensel-Hammes P Bajjalieh SM Matagne A The synaptic vesicle protein SV2A is the binding site for the antiepileptic drug levetiracetam. Proc Natl Acad Sci U S A (2004) 101:9861–6. 10.1073/pnas.0308208101 15210974 PMC470764

[40] Yang XF Weisenfeld A Rothman SM. Prolonged exposure to levetiracetam reveals a presynaptic effect on neurotransmission. Epilepsia (2007) 48:1861–9. 10.1111/j.1528-1167.2006.01132.x 17521346

[41] Lukyanetz EA Shkryl VM Kostyuk PG. Selective blockade of N-type calcium channels by levetiracetam. Epilepsia (2002) 43:9–18. 10.1046/j.1528-1157.2002.24501.x 11879381

[42] Girardeau G Benchenane K Wiener SI Buzsaki G Zugaro MB. Selective suppression of hippocampal ripples impairs spatial memory. Nat Neurosci (2009) 12:1222–3. 10.1038/nn.2384 19749750

[43] Ego-Stengel V Wilson MA. Disruption of ripple-associated hippocampal activity during rest impairs spatial learning in the rat. Hippocampus (2010) 20:1–10. 10.1002/hipo.20707 19816984 PMC2801761

[44] Lopez-Gongora M Martinez-Domeno A Garcia C Escartin A. Effect of levetiracetam on cognitive functions and quality of life: a one-year follow-up study. Epileptic Disord (2008) 10:297–305. 10.1684/epd.2008.0227 19017572

[45] Javed A Cohen B Detyniecki K Hirsch LJ Legge A Chen B Rates and predictors of patient-reported cognitive side effects of antiepileptic drugs: an extended follow-up. Seizure (2015) 29:34–40. 10.1016/j.seizure.2015.03.013 26076842

[46] Lancman ME Fertig EJ Trobliger RW Perrine K Myers L Iyengar SS The effects of lacosamide on cognition, quality-of-life measures, and quality of life in patients with refractory partial epilepsy. Epilepsy Behav (2016) 61:27–33. 10.1016/j.yebeh.2016.04.049 27315132

[47] Schoenberg MR Rum RS Osborn KE Werz MA. A randomized, double-blind, placebo-controlled crossover study of the effects of levetiracetam on cognition, mood, and balance in healthy older adults. Epilepsia (2017) 58:1566–74. 10.1111/epi.13849 28731266

[48] IJff DM van Veenendaal TM Majoie HJ de Louw AJ Jansen JF Aldenkamp AP. Cognitive effects of lacosamide as adjunctive therapy in refractory epilepsy. Acta Neurol Scand (2015) 131:347–54. 10.1111/ane.12372 25630655

[49] Deshpande LS Delorenzo RJ. Mechanisms of levetiracetam in the control of status epilepticus and epilepsy. Front Neurol (2014) 5:11. 10.3389/fneur.2014.00011 24550884 PMC3907711

